# Invasive lobular carcinoma of the breast: morphology, biomarkers and ’omics

**DOI:** 10.1186/s13058-015-0519-x

**Published:** 2015-01-30

**Authors:** Amy E McCart Reed, Jamie R Kutasovic, Sunil R Lakhani, Peter T Simpson

**Affiliations:** The University of Queensland, UQ Centre for Clinical Research, Herston, QLD 4029 Australia; Pathology Queensland, The Royal Brisbane & Women’s and Gold Coast Hospitals, Herston, QLD 4029 Australia; School of Medicine, The University of Queensland, Herston, QLD 4006 Australia

## Abstract

Invasive lobular carcinoma of the breast is the most common ‘special’ morphological subtype of breast cancer, comprising up to 15% of all cases. Tumours are generally of a good prognostic phenotype, being low histological grade and low mitotic index, hormone receptor positive and HER2, p53 and basal marker negative, and with a generally good response to endocrine therapy. Despite this, clinicians face countless challenges in the diagnosis and long-term management of patients, as they encounter a tumour that can be difficult to detect through screening, elicits a very invasive nature, a propensity for widespread metastatic colonisation and, consequently, in some studies a worse long-term poor outcome compared with invasive carcinoma of no special type. Here we review the morphological and molecular features that underpin the disparate biological and clinical characteristics of this fascinating tumour type.

## Introduction

Invasive lobular carcinoma (ILC) is the most common ‘special’ type of breast cancer and presents with a distinct morphology and clinical behaviour compared with invasive carcinoma of no special type (IC-NST). Typically, ILC tumours display features associated with a good prognosis, being low grade and oestrogen receptor positive; however, the tumour can be highly metastatic [1] and several studies demonstrate that the overall long-term outcome for patients diagnosed with ILC may be similar or worse than for patients diagnosed with IC-NST [2,3]. E-cadherin loss is responsible for the inherently discohesive phenotype associated with ILCs, and changes at the genomic level account for this loss. Recent technological advances have generated masses of genomic and transcriptomic data, some of which is further illuminating the natural history of ILCs. We present here a review of lobular carcinoma, paying particular attention to the morphological and immunophenotypic features of pre-invasive and invasive lesions, the importance of E-cadherin dysfunction in tumour biology, transcriptomics, genomics and diagnostic aspects that aid patient management.

## Morphological characteristics of lobular neoplasia and invasive lobular carcinoma

**Figure 1:**
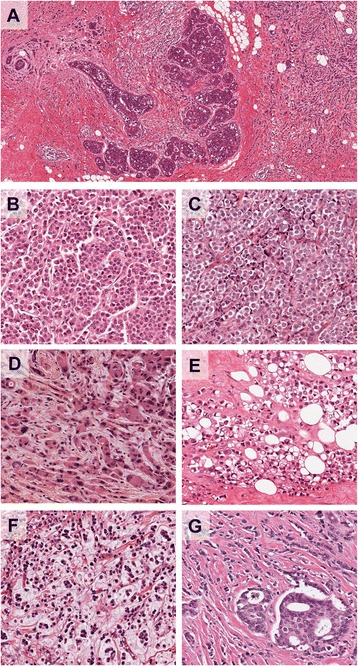
**Morphological characteristics of invasive lobular carcinoma and its variants. (A)** Low power view of a terminal duct lobular unit colonised by lobular carcinoma *in situ.* Classic invasive lobular carcinoma is seen diffusely infiltrating the whole specimen as single cells and single files of cells. The characteristic targetoid growth pattern is evident on the left-hand side (see also Figure 2). **(B-G)** Morphological variants of the classic type: **(B)** alveolar type, with globular aggregates of approximately 20 cells; **(C)** solid type with discohesive tumour cells growing in solid sheets; **(D)** a pleomorphic variant - note the pink, foamy cytoplasm typical of an apocrine phenotype and irregular nuclei; **(E)** pleomorphic invasive lobular carcinoma with prominent signet ring cells; **(F)** invasive lobular carcinoma showing mucinous/histiocytoid morphology; **(G)** mixed ductal-lobular carcinoma.

**Figure 2:**
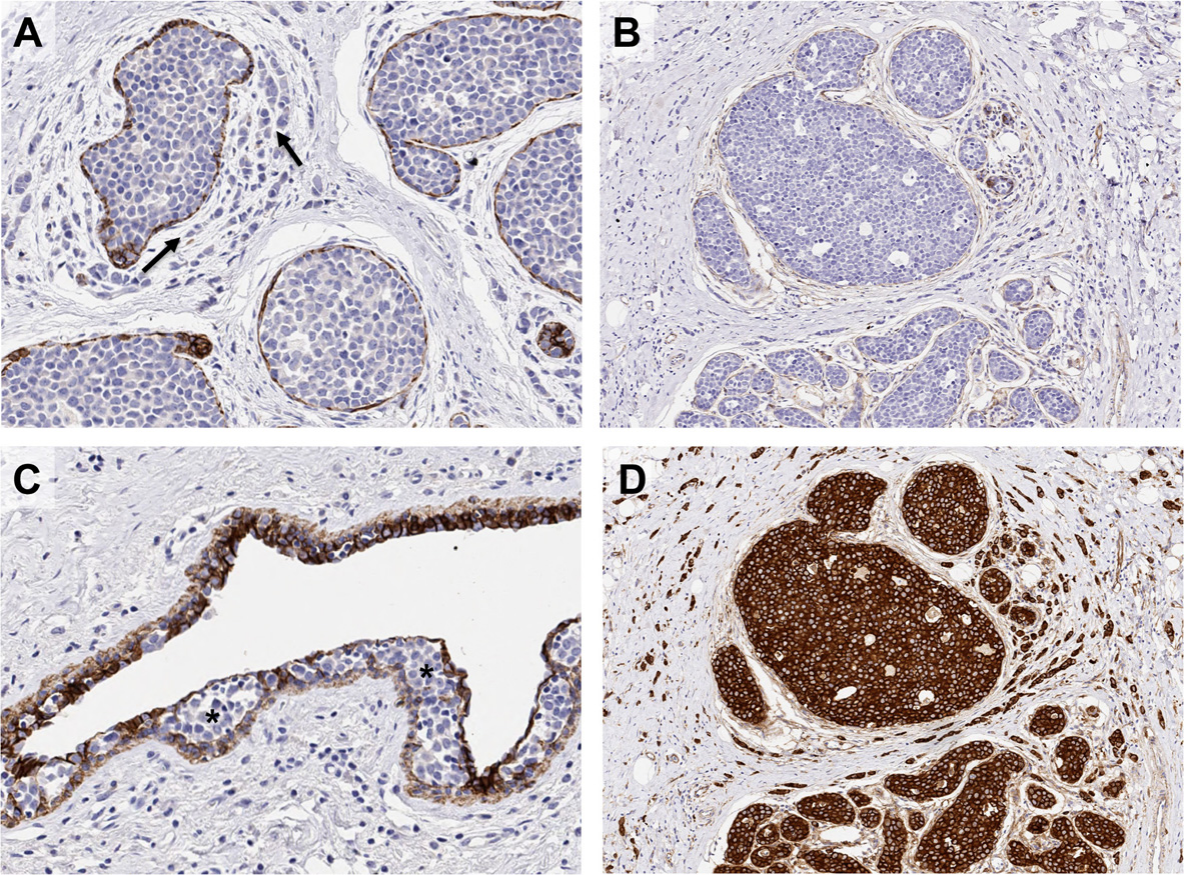
**Immunohistochemical staining of E-cadherin and its binding complex in invasive lobular carcinoma.** Lobular carcinoma *in situ* (LCIS) and invasive lobular carcinoma (ILC); note invasive neoplastic cells of ILC (arrows) growing in a targetoid fashion around the *in situ* component. **(A)** E-cadherin and **(B)** β-catenin staining is negative in both LCIS and ILC, although positive staining is observed in the myoepithelial cells surrounding the LCIS. **(C)** Pagetoid spread (asterisks) is also observed in this case, whereby the neoplastic cells (negative for E-cadherin) are growing and invading between the luminal and myoepithelial cells of a normal ductal structure). **(D)** In the absence of E-cadherin, there is a strong re-localisation of p120-catenin to the cytoplasm of neoplastic cells in LCIS and ILC.

Diagnostic criteria for lobular neoplasia (LN) and ILC (Figure 1) are now well established and described [4] and so are only briefly outlined below. The term ‘lobular neoplasia’ was introduced [5] to encompass a spectrum of *in situ* neoplastic proliferations including atypical lobular hyperplasia (ALH) and lobular carcinoma *in situ* (LCIS), which describe different levels of involvement of individual lobular units. The descriptions ALH and LCIS are widely used to classify these lesions since they confer different relative risks (4- to 5-fold and 8- to 10-fold, respectively) for the patient to subsequently develop invasive cancer compared with the general population [6]. By definition, neoplastic cells of LN remain confined to the terminal duct-lobular unit, but they may exhibit pagetoid spread in which cells can migrate along the ductal system between the basement membrane and normal epithelial cell population (Figure 2).

The cells of LN and ILC are typically small, monomorphic and lack cohesion, with round or notched ovoid nuclei and a thin rim of cytoplasm. Intra-cytoplasmic lumen, containing a central inclusion of mucin, may be present and in some cells this may be large enough to create a signet ring cell-type appearance (Figure 1). Cells of classic LCIS or ILC may vary in appearance and have been referred to as type A cells (classic) or the larger type B cells (vesicular nuclei) that may show mild pleomorphism. Cells of the pleomorphic type of LCIS (PLCIS) may be larger still and exhibit marked nuclear pleomorphism, akin to that observed in high-grade ductal carcinoma *in situ* (DCIS) [7]. Extensive or florid LCIS is also important to recognize. These lesions are characterized by the proliferation of the same type A or type B cells, but there is marked expansion of involved lobular units and areas of necrosis and microcalcification [8].

In classical ILC, the characteristic pattern of growth involves the infiltration of single cells or single files of cells through the stroma, with little disturbance of normal tissue architecture. The invading tumour cells are frequently arranged in a concentric (targetoid) pattern around normal ducts or structures (Figure 2). There are a series of morphologically recognised variants that demonstrate either cytological or architectural variation of the characteristic features of classic ILC. Pleomorphic lobular carcinoma (PLC) retains the distinctive growth pattern of classic ILC but, as in its *in situ* counterpart (PLCIS), there is marked cellular atypia and nuclear pleomorphism relative to classic LN and ILC. PLC may also have an increased mitotic rate, be composed of signet ring cells (Figure 1) and/or show apocrine or histiocytoid differentiation. Conversely, the solid and alveolar variants are both characterized by classic ILC cells (small, regular sized and lacking cohesion) that are arranged in sheets (solid type) or in aggregates of at least 20 cells (alveolar type, Figure 1) rather than in single cords of cells. Solid ILC may also be more frequently pleomorphic and mitotically active relative to classic ILC. Classic ILC may be admixed with one or more of these morphological variants or with tumour cells of a tubular growth pattern (tubulo-lobular carcinoma). Furthermore, around 5% of all invasive breast tumours exhibit mixed features of both ductal and lobular differentiation [4,9] (Figure 1).

Histological grading is an important part of breast tumour classification, and is performed using the Nottingham histological grading system. There is debate, however, as to the relevance of this system for the ‘special types’ including lobular carcinomas and some studies suggest it is of limited value since tubule formation is rare (except in the tubulo-lobular variant), there is limited nuclear pleomorphism and the mitotic count is frequently low. Consequently, most ILCs, including variants, are grade 2. Nevertheless, other studies report that grade is indeed an independent prognostic factor in ILC, as it is in breast cancer in general, with mitotic count being the most useful component for predicting poor outcome [10]. Furthermore, while several studies report that morphological variants are aggressive subtypes associated with poor outcome, particularly relative to classic type [11], evidence suggests that a nuclear pleomorphism score of 3 (which would indicate a classification of PLC), in an overall grade 2 tumour does not add prognostic value, the most important discriminator being overall grade and/or mitotic count [12].

ALH, LCIS and PLCIS can be frequently found co-localised in the same specimen, also alongside other non-obligate precursors such as columnar cell lesions, atypical ductal hyperplasia and low-grade DCIS as part of the ‘low-grade’ family of breast precursor lesions [13]. LNs frequently co-exist with invasive carcinomas of lobular type, including classic ILC (Figures 1 and 2) and tubulo-lobular carcinomas (in 90% and 57% of cases, respectively [13]), supporting a common evolutionary origin of these lesions. Indeed, the overlapping cytological appearance and frequent co-localisation of LN and ILC, combined with concordant immunophenotypic and molecular characteristics, supports the notion that LCIS and PLCIS are clonal and non-obligate precursor lesions for ILC and PLC, respectively [14,15].

## Immunophenotyping invasive lobular carcinoma

Classic ILCs are almost always hormonally regulated. Up to 95% of cases express oestrogen receptor (ER)α and 60 to 70% of cases express progesterone receptor [2,16,17], whereas only 60 to 70% of IC-NST express these two biomarkers. ERα is always expressed in the alveolar variant (100%) yet is less frequently found in pleomorphic ILC (10 to 76%) [10,18]. The androgen receptor and ERβ are also expressed in approximately 90% of ILCs [10,19]. The interplay between these receptors in ILC is yet to be fully elucidated, though it is clear the high frequency of hormone receptor expression reflects the overall good response to endocrine-based therapies [2].

Biomarkers associated with poor clinical behaviour are rarely expressed in ILC, including the HER2, p53 and basal/myoepithelial markers (cytokeratins 14 and 5/6, epidermal growth factor receptor, smooth muscle actin and p63) [10,16,17]. Generally the proliferation index (measured by Ki67 staining) is low in ILC, reflecting the low mitotic count (see above) and this likely contributes to reduced response to chemotherapy relative to patients diagnosed with IC-NST. Pleomorphic ILCs, on the other hand, are more likely to exhibit HER2 amplification (in 35 to 80% of cases) and p53 expression and the proliferation index is typically higher [10,18].

## E-cadherin dysfunction - master regulator of the lobular phenotype

The characteristic discohesive growth pattern of ILC is the result of the dysregulation of cell-cell adhesion properties, primarily driven by the targeted disruption of the cell adhesion molecule E-cadherin. E-cadherin is a calcium-dependent transmembrane protein that mediates cell-cell adhesion and cellular polarity by binding to itself on neighbouring cells in a homophilic manner. The intracellular domain of E-cadherin associates with the actin cytoskeleton via α-, β-, γ- and p120 catenins to form adherens junctions between non-neural epithelial cells. E-cadherin is largely regulated by its catenin-binding partners, which anchor E-cadherin to the membrane and the actin cytoskeleton. E-cadherin-mediated cell adhesion maintains cell viability and when this adhesion is lost the detached cells undergo a cell death program called anoikis.

In normal breast epithelial cells and in most IC-NST, E-cadherin and the associated catenin binding proteins are located at the cell membrane, maintaining cellular cohesion. In contrast, approximately 90% of LNs and ILCs, including variants, completely lack E-cadherin protein expression [15,20-23]. The loss of E-cadherin in ILC also results in the loss of α-, β- and γ-catenins, and p120-catenin becomes up-regulated and re-localised to the cytoplasm [24]. From a biological point of view, this re-localisation of p120 has been found to enable anoikis resistance in lobular cells, allowing them to survive independently of attachment to neighbouring epithelial cells and promote cell migration through activation of Rho/Rock signalling [25]. E-cadherin expression has become an important diagnostic feature of LN and ILC and the cytoplasmic localisation of p120-catenin is a positive immunohistochemistry marker for ILC [26]. In combination these biomarkers may aid in classification when it is difficult to differentiate between lobular and ductal lesions [26]; however, there remains confusion regarding the interpretation and so caution is warranted. In particular it is important to remember that approximately 10% of ILCs still express E-cadherin [10,22], either with normal membrane localisation or aberrantly distributed as fragmented membrane and/or cytoplasmic staining. The E-cadherin-catenin complex may be dysfunctional in these cases due to the presence of *CDH1* gene mutation (see below) or aberrant/loss of expression of the catenin binding proteins [22], which may be detected using β-catenin and p120-catenin immunohistochemistry. However, a diagnosis of LN or ILC based on morphologic and cytologic criteria should therefore not be reclassified as DCIS or IC-NST based on the status of these immunohistochemical markers [26].

E-cadherin deregulation occurs in the earliest morphological stage of lobular tumourigenesis (that is, ALH) and is frequently and irreversibly driven by genomic alterations targeting its gene, *CDH1* (located at chromosome 16q22.1). Molecularly, the patterns of E-cadherin loss often follow Knudsen’s two hit hypothesis for a classic tumour suppressor gene, involving *CDH1* mutation, gene methylation and/or loss of heterozygosity in the region of 16q22.1 (frequently involving the whole chromosomal arm).

Promoter hypermethylation and concomitant down-regulation of *CDH1* expression has been reported in 21 to 77% of ILCs [27,28] and the detection of methylated *CDH1* promoter sequences in adjacent normal tissues and in LN implies that this is an early hit [29]. The somatic copy number loss of 16q in ILC and ER-positive, low-grade IC-NST is extremely frequent, suggesting these tumours share a common pathway of evolution. We reviewed the DNA copy number status at the *CDH1* gene locus in the 153 lobular tumours from The Cancer Genome Atlas (TCGA) data resource [30,31] and this revealed that 12.4% of tumours show a diploid copy number; 84.3% show a single copy loss and 3.3% show a putative homozygous deletion. Chromosomal analysis of LNs has shown they too lose chromosome 16q [8,32-34], suggesting this is also an early assault on the *CDH1* gene region.

**Figure 3:**
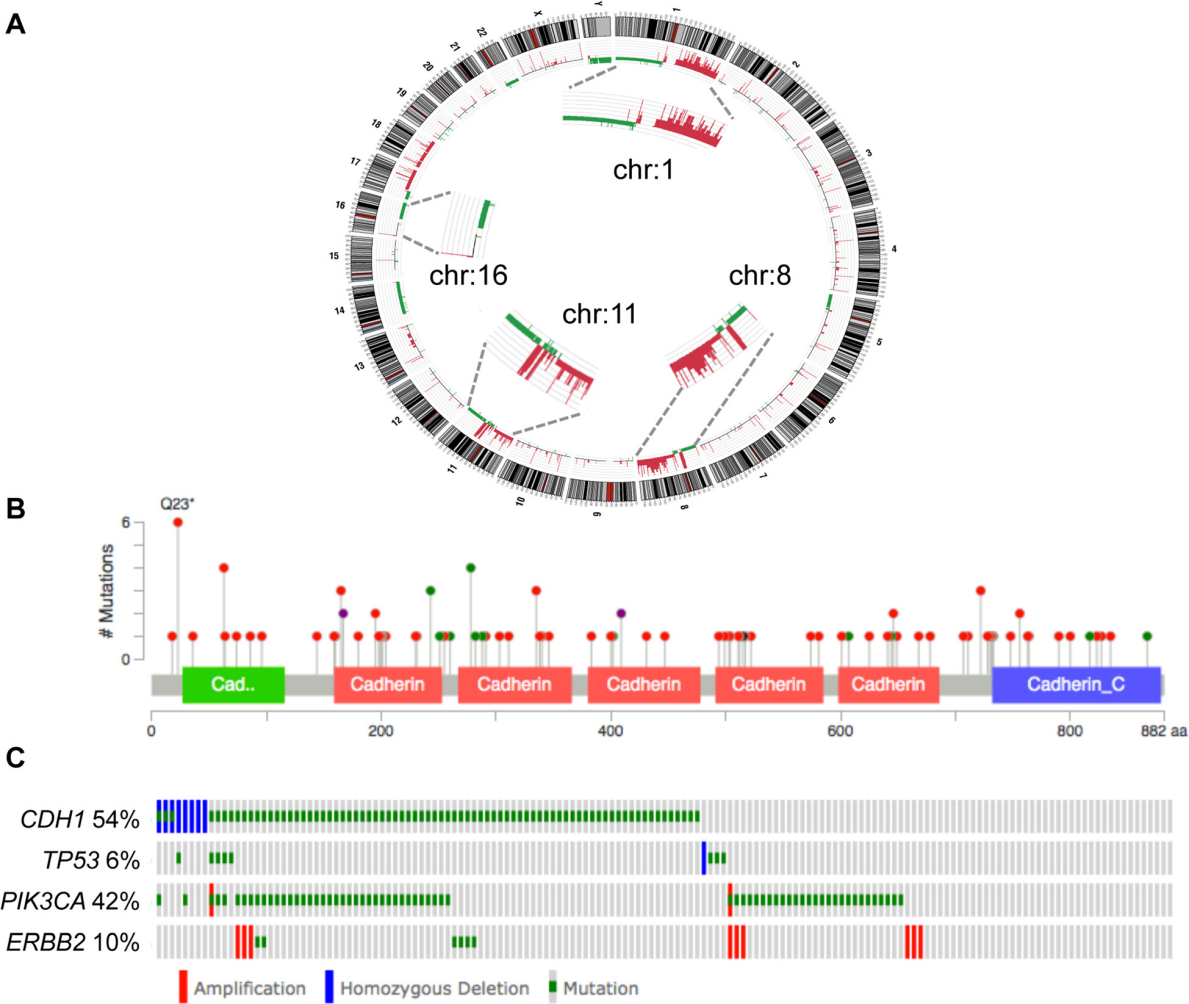
**Genomic architecture of invasive lobular carcinoma. (A)** CIRCOS plot of an invasive lobular carcinoma (ILC) tumour profiled using the Illumina Omni 2.5 million SNP CNV array. Note the archetypal ILC changes, including chromosome 1q gain, 8p amplification, 11q13 amplification and 16q deletion. **(B)** Spectrum of somatic mutations across the E-cadherin coding region in the cBioPortal ILC data set [30,31]. Note the cadherin prodomain in green and the cadherin cytoplasmic domain in blue; missense mutations in green and nonsense mutations in red. **(C)** Oncoprint depicting the frequency of somatic mutations in key, recurrently altered cancer genes (*CDH1*, *TP53*, *PIK3CA*, *ERBB2*) affecting 75% of the 155 ILCs in The Cancer Genome Atlas cohort [30,31]. Percentages are numbers of tumours exhibiting an alteration in the specified gene.

Somatic mutations are found dispersed throughout the *CDH1* coding region and are frequently truncating [21] (Figure 3). Identical *CDH1* genetic mutations have been detected in LCIS and in their adjacent invasive counterpart [15], which is a key finding implicating LCIS as a direct (but non-obligate) precursor for ILC. Further to this, *CDH1* mutations were detected in LCIS [35], although, surprisingly, no mutations were found in adjacent, microdissected ALH lesions. This may be a question of technological sensitivity and so the application of high-resolution massively parallel sequencing technologies is certainly warranted to clarify such findings.

The reported frequency of *CDH1* mutation and loss of heterozygosity is unexpectedly discrepant between studies (from 30 to 80%) [10,21,36]. Improving technologies and increasing cohort sizes have not necessarily resolved this. For example, TCGA [37] reported from an exome sequencing strategy (that is, enriching for exons only) that *CDH1* mutations were very common (30/36; 83%) within the lobular histological subtype and, expectedly, corresponded with low E-cadherin expression. TCGA resource has now made comprehensive ’omic data available for 958 breast cancers through the cBioPortal [30,31] and in an investigation of these data we identified *CDH1* mutations in 78 out of 155 ILCs (50%). This latter figure is supported by an independent exome sequencing study of ER-positive tumours in the clinical context of aromatase inhibitor response, where they identified *CDH1* mutation in 20 out of 40 ILCs [38].

Mutations in *CDH1* have also been identified in other types of epithelial cancers, most notably in diffuse gastric carcinomas, which have a very similar infiltrative growth pattern to ILC of the breast. Hereditary diffuse gastric carcinoma is sometimes caused by a germline mutation in *CDH1* [39] and mutation carriers have an increased risk for developing ILC. A diagnosis of ILC may also be enriched within breast cancer families and since LNs/ILCs more frequently present as multifocal or bilateral disease, it fits with a theory of a germline predisposition to tumour development. Despite E-cadherin being the obvious candidate for such a predisposition, early work suggested *CDH1* germline variants are rare in familial lobular breast cancer [40] but do account for some cases of bilateral ILC [41]. Considerable evidence arising from studying the human disease therefore exists for E-cadherin playing a major role in the initiation and biology of both lobular and diffuse gastric cancer. Animal models of hereditary diffuse gastric cancer and lobular breast cancer provide additional support for this concept, whereby *CDH1* germline deficiency in combination with a second hit (carcinogen treatment or *TP53* mutation) is sufficient to initiate disease development [42,43]. (These aspects are covered in more detail in a review in this series [44].)

The loss of E-cadherin is also associated with the process of epithelial to mesenchymal transition (EMT) where cells lose polarity and adhesion to become more migratory and invasive during embryonic morphogenesis and wound healing. Tumour cells are believed to be able to hijack this process to facilitate migration away from the primary tumour microenvironment and metastatic dissemination. The acquisition of the mesenchymal phenotype is accompanied by cadherin switching (loss of E-cadherin and activation of N-cadherin), which is driven by transcriptional regulators of E-cadherin, including SNAIL and TWIST, as well as post-transcriptionally active microRNAs (for example, the miR200 family), and the gain in expression of mesenchymal markers, such as vimentin. Given the loss of E-cadherin and the infiltrating growth pattern of ILC, it is tempting to speculate that EMT plays a mechanistic role in driving this phenotype. Indeed, a meta-analysis of microarray gene expression data found TWIST to be highly expressed in human ILC samples, showing 70% had elevated TWIST mRNA expression, compared with 32% of ductal carcinomas [45]. However, immunohistochemical analysis of EMT markers in human breast tumours demonstrated that: i) neoplastic lobular cells retain their epithelial identity; ii) TWIST protein was expressed by the fibroblasts in the prominent stromal component of ILC; and iii) only 1 out of 24 (4%) ILCs expressed EMT markers [46]. While EMT is traditionally associated with late stages of tumour progression (invasion and metastasis) and is a dynamic process, the loss of E-cadherin in ILC is an early and typically irreversible event in ILC. Thus, the functional role of EMT in driving the invasive nature of ILC remains unlikely.

## Transcriptome profiling of lobular tumours

At the turn of the century, a pivotal study used gene expression profiling microarrays to categorise breast cancers into a series of ‘intrinsic’ subtypes that stratified prognosis: luminal A, luminal B, HER2, and basal-like [47,48]. These categories have since been expanded to include claudin-low [47,48] and normal breast-like. Due to the nature of ILCs generally being low-grade and ER-positive, they are frequently classed as luminal A, and owing to their commonly infiltrative histology and thus a comparatively reduced tumour to stroma cellularity (compared to ductal tumours), they may also be classed as normal-like, simply as a consequence of there being more normal cells and/or stroma in their processed samples [49]. Ultimately though, like ductal carcinomas, they are a heterogeneous group and have the potential to be classed as any of the defined subtypes, including molecular apocrine for the PLC variant [48,50], while, interestingly, the non-lobular special types of breast cancer (for example, medullary, metaplastic, micropapillary, tubular, apocrine and neuroendocrine carcinomas) cluster within a single subtype only, underscoring their more inherent homogeneity.

Gene expression profiling studies have also been undertaken to better understand the biological differences between lobular and ductal invasive tumours. Overall, the number of lobular tumours profiled has been considerably lower than that for ductal invasive tumours [51-55]). Korkola and colleagues [52] defined 11 genes as capable of differentiating ILCs from ductal carcinomas, but more recent studies report larger, functional groups of genes as being responsible for their different aetiologies. Of most relevance are those functional gene groups that were identified when 20 ILCs were compared with 91 ER-positive, grade-matched invasive ductal carcinomas (IDCs): adhesion, transforming growth factor beta signalling; cell communication and trafficking; actin remodelling; lipid/prostaglandin synthesis; transcription factor/immediate early genes [54]. Ultimately, other than expected transcriptional changes associated with E-cadherin dysfunction, at the individual transcript level there was minimal overlap between all five studies. Given the variety of platforms used for these assays, small sample sizes and modes of analysis, this is not altogether surprising. A meta-analysis of these studies identified *THBS4* (thrombospondin 4) as a potential ILC biomarker, but investigations at the protein level confirmed no difference in expression between ILCs and their ductal counterparts, and instead revealed THBS4 as a marker of tumour-associated extracellular matrix [56]. Again, this finding is probably more associated with the fact that ILC tumours exhibit a higher stromal content, thus skewing the subsequent downstream analyses.

## The genomic landscape of lobular carcinomas

LNs and ILCs are more likely to be diploid than ductal tumours [16]. Indeed, chromosomal and array-based comparative genomic hybridisation (aCGH) analyses have defined, on a gross scale, the genomic profile of lobular carcinomas - in short, they harbour fewer chromosomal changes than ductal carcinomas and are generally less complex. Genomic losses, such as at 16p, 16q, 17p and 22q, and gains at 6q were detected in LN by chromosomal CGH [33]. The key alterations identified more recently by aCGH in classic LCIS, florid/extensive LCIS and PLCIS are 1q gain and 16q loss, with increased genomic complexity observed in the latter two groups of lesions, including loss of 8p, 11q and 17p and amplifications at 11q13 (*CCND1*) and 17q12 (*ERBB2*) [8,14,34]. Like their pre-invasive counterparts and ER-positive IC-NST, both classic and pleomorphic ILC exhibit a high frequency of gain of chromosome 1q and loss of 16q [18,23,57,58] and it has been reported that all ILCs lose at least part of 16q [58]. Other recurrent alterations include losses at 8p23-p21, 11q14.1-q25, and 13q, gains of 8q and 16p, and high-level amplifications at 1q32, 8p12-p11.2, and 11q13. Although some candidate genes in the various regions have been postulated (for example, *FGFR1* in 8p12-p11.2 and *CCND1* in 11q13 [23]), no definitive data confirming the drivers contained in these various regions have been reported specifically for lobular breast cancer. This is likely a result of the complexity of the chromosomal changes and the context-dependent nature of some of these alterations. Numerous candidate oncogenes have been identified in these regions but not specifically for lobular tumours - for example, *ZNF703* gene amplification at 8p12 specifies luminal B breast cancer [59]. As mentioned above, PLC contains a similar profile of chromosomal change, although there is increased complexity and additional amplifications are present - 8q24 (*MYC*), 17q12 (*ERBB2*/Her2) and 20q13, which are usually considered to be archetypal changes of high-grade ductal tumours [18]. Some attempts have been made to classify tumour genome profiles based on genomic architecture as either simple, complex-firestorm or complex-sawtooth. The genomes of both classic and pleomorphic ILC are generally classified as simple (in that they frequently harbour 1q gain and 16q loss and few other alterations) or complex-firestorm (relating to the additional presence of complex, high-level amplifications at the stated loci) [18,23]. It is conceivable that those ILCs that are classed as complex-firestorm have a worse prognosis, though this has yet to be explored.

A catalogue of the transcriptomic and genomic architecture of 2,000 breast cancers, and their integration into novel clusters was reported in 2012 [60]. The discovery set of this large study included 148 classic ILCs, of which 88.5% were ER positive and were classified as: luminal A, 44.9%; luminal B, 19.7%; basal, 2.7%; HER2, 6.1%; normal, 25.9%. This study also presented an alternative categorisation algorithm combining transcriptome and genomic copy number data to define 10 ‘integrative clusters’ (IntClusts). ILCs were predominantly assigned to IntClust 3 (39.7%), 4 (23.5%) and 8 (14.7%), with varying frequencies of the archetypal chromosomal changes (1q+, 16p+, 16q-). Predictably, IntClust 3, into which most ILCs segregated, showed overall the simplest genomes, a high frequency of 1q + and 16q- and the best survival outcome. Similarly, tumours in IntClust 8 also harbour a high frequency of 1q + and 16q-, but also 16p+. Conversely, tumours in IntClust 4 showed infrequent 1q + and 16q-. The groups in which lobular carcinomas are not enriched (that is, less than approximately 6% of the ILCs) showed more recurrent gains/amplifications on chromosomes 8q, 11q or 17q. Subtle variation in the genomic alterations in these tumours may therefore have a strong influence on tumour behaviour.

### The data era: ‘next-generation’ sequencing

Significant technological advances in recent years have meant that the depth of interrogation of individual tumour genomes has increased significantly. This so-called ‘next-generation sequencing’ technology combined with the activities of several large consortia has led to the production of masses of high quality sequence and genomic copy number data for large numbers of tumours. As noted above, two studies have performed exome sequencing on ILC of any significant numbers [37,38]. Beyond the highly recurrent mutations in *CDH1* and *PIK3CA*, which for *PIK3CA* the mutation rate is similar to that observed overall in ER-positive breast cancers, there is a paucity of recurrent driver mutations in this tumour type (Table 1), supporting the idea that heterogeneity within and between tumours is complex.

**Table 1:** **Recurrent mutations in invasive lobular carcinoma**

**Gene**	**cBioPortal** [30,31] **(n = 155)**	**Ellis** ***et al.*** [38] **(n = ~40)** ^**a**^	**Ross** ***et al.*** [62] **(n = 22)** ^**b**^
***AKT1***	0.6%	NR	9.1%
***ARID1A***	4.5%	NR	NA
***ATR***	2.0%	2.5%	NA
***BIRC6***	2.0%	2.5%	NA
***CDH1***	50.0%	50.0%	100%
***CDKN1B***	1.3%	3.4%	NA
***ERBB2***	3.8%	NR	18%
***GATA3***	2.6%	3.4%	NA
***KRAS***	0.6%	NR	9.1%
***MALAT1***	0.0%	2.5%	NA
***MAP2K4***	0.6%	3.4%	9.1%
***MAP3K1***	5.0%	13.8%	NA
***NCOR1***	4.5%	9.1%	NA
***NF1***	3.2%	NR	NA
***PIK3CA***	41.0%	41.4%	36.4%
***RB1***	1.30%	0	9.1%
***RUNX1***	7.0%	5.0%	4.5%
***SMAD4***	1.30%	NR	NA
***TP53***	5.0%	12.1%	36.4%

^a^The exact number of invasive lobular carcinomas in the Ellis study is difficult to define as several cohorts were assessed using a variety of technologies. ^b^The Ross study selected only *CDH1* mutant cases. NA, not available; NR, not reported.

One of the first studies to report the application of the then novel sequencing technologies to breast cancer samples was that of Shah and colleagues in 2009 [61]. This study sequenced a pleural effusion metastasis and matched primary ILC diagnosed 9 years earlier and found that 5 somatic mutations (of a possible 32 defined for the metastasis) were present in the primary tumour, a telling comment on the degree of clonal evolution occurring during progression from primary clone to metastasis. This patient also had an *ERBB2* mutation, as did 2 of 192 ILCs in their validation set. Somatic mutations (not including amplifications) in *ERBB2* have since been shown to be generally rare in breast cancer but interestingly were significantly enriched in the ILC subtype [37]. Consulting the cBioPortal [30,31] for an updated data review, 6 of 155 ILCs (3.9%) harboured an *ERBB2* mutation. Interestingly, in a massively parallel, targeted amplicon sequencing of ‘actionable cancer genes’ in ILC post-treatment relapse (that is, recurrence or metastasis), Ross and colleagues [62] reported *HER2*/*ERBB2* genetic alterations in 6 of 22 (27%) cases, including 4 mutations, one gene fusion and one amplification. HER2 is an important clinically actionable target, indicating this type of targeted sequencing analysis, which has greater sensitivity than exome sequencing and is applicable to formalin-fixed paraffin-embedded tissue, may soon aid in the management of patients when planning primary or secondary treatment regimes.

## Diagnostic algorithms

As the era of molecular technology for subtyping disease and identifying molecular targets takes huge leaps forward it is tempting to begin to ignore the more traditional morphological classification of disease and consider molecular subtyping (for example, luminal, basal, HER2 subtypes) and testing (for example, OncotypeDX) for classification and management. However, the breast cancer morphological special types remain fundamental to the management of patients since the biological and clinical significance of these entities provides important information regarding appropriate management strategies and outcomes.

A diagnosis of lobular carcinoma, as a special morphological type, supports this idea, since there are clinical and biological features that set it apart from the more commonly diagnosed IC-NST, and despite the ‘good prognostic features’ exhibited by ILC, some large studies consistently demonstrate that ILCs have a similar or worse long-term outcome compared with IC-NST [2,3]. Many of the challenging aspects in the diagnosis and management of ILC relate to the indolent but highly infiltrative nature of the tumour growth and the biology of dysfunctional E-cadherin that underpins this. For instance, LNs and ILCs are not always detected as a palpable mass and can be difficult to detect by mammography [63] owing to the rare association with calcification and the lack of host stromal response to the diffusely infiltrating tumour.

Differentiating classic LCIS from its morphological variants (that is, extensive/florid LCIS and PLCIS) may be important from a management point of view, owing to anecdotal evidence that these lesions have a different clinical course and that they exhibit more genomic instability [8,14,34]. Correct diagnostic classification of LN is also very important because the management of patients diagnosed with LN are different to those with DCIS, in the setting of core needle biopsy or surgical margin status, where further excision is required for all cases of DCIS but not for LN. There is a significant body of literature regarding this and readers are directed to [10,26,64] and references therein for more information. Briefly, differentiating LCIS and PLCIS from low- and high-grade DCIS, respectively, or lesions with indeterminate features may be difficult in certain scenarios. The use of ancillary immunohistochemical staining for E-cadherin, β-catenin and p120-catenin can therefore be helpful to aid classification [24,26]. In terms of ILC, histological grading is considered a critical component of classification, and description of the morphological variants is recommended given the prognostic insight this may provide and the potential for future epidemiological and biological studies related to tumour subtyping [11,12,65]. As above, the use of E-cadherin, p120 catenin or β-catenin is appropriate to help resolve the diagnosis of difficult cases, although it is important to consider classification first based on morphology and cytology and not to reclassify a *bona fide* ILC as IC-NST based on ‘normal’ E-cadherin or p120-catenin staining since around 10% of ILCs still express membranous E-cadherin [20,22,26]. Pan-cytokeratin markers are also utilized to differentiate small ILC cells from macrophages in biopsies and extremely diffuse cases.

ILCs respond less well to chemotherapy compared to IC-NST, probably resulting in part from their indolent, low proliferative index (low mitotic count and low Ki-67 index). Many molecular tests are now available to prognosticate and inform decisions regarding the addition of chemotherapy to a patient’s treatment program. Many ILC tumours meet the requirements for the Oncotype DX 21-gene clinical assay, in that they are generally grade 2 and ER positive, and may not have spread to the lymph nodes. The usefulness of this and other tests is reviewed in [66], where it is also noted that many of these signatures focus on proliferation as a mechanism of assessing likelihood of recurrence.

Expression of ER, progesterone receptor and HER2 guide therapeutic decisions and the vast majority of patients will receive endocrine-based therapy, for which there is generally a good response [2]; however, *de novo* or acquired resistance is an inevitable problem for some patients. The somatic mutation profile of a tumour may contribute to this; for example, tumours harbouring or acquiring driver mutations in *ESR1* [67] or *ERBB2* [37] or amplifications at 8p12 (*FGFR1*) or 11q13 (*CCND1*) [23] may be less responsive to targeted endocrine therapy. In support of this, the ER-positive ILC cell line model MDA-MB-134VI was found to be *de novo* tamoxifen resistant, yet cells were sensitized to anti-oestrogen therapy when in combination with FGFR1 inhibitors [68]. Oestrogen-related receptor gamma/AP1 signalling may also mediate tamoxifen resistance in the SUM44 cell model system [69]. Recent research has also shown that *PIK3CA* mutations are selected for during progression from the primary ILC tumour to a local recurrence but not through to dissemination of distant metastases [70]. While links between *PIK3CA* mutation and endocrine therapy resistance have been investigated in some breast cancers, this mechanism has not been specifically studied in ILC; however, it is reasonable to hypothesize that this may be the case in some endocrine-resistant ILCs [71]. A gene expression study comparing ILC and IDC tumour biopsies in the neo-adjuvant setting suggests that letrozole both induces near identical transcriptome changes in the tumour types and does not interfere with histological subtype-specific gene expression [72]. Recent data suggest there may be an improved response to the aromatase inhibitor letrozole compared with tamoxifen in ILCs but the biological mechanisms driving the differences in response need to be further investigated [73]. As our understanding of the biological mechanisms that underpin response and resistance to anti-oestrogen therapies improves we will be able to better predict which treatment regime would be most effective (endocrine therapy or in combination with other targeted therapies).

## Conclusion

Lobular carcinoma is an important breast cancer subtype with some peculiar clinical and biological characteristics compared with the more commonly diagnosed IC-NST. Rather surprisingly, and despite the good prognostic features of the primary tumour and good response to endocrine therapy, the long-term outcome for patients diagnosed with ILC is, in some studies, worse than for IC-NST. There remain significant challenges, therefore, managing patients with this specific disease. Although considered a ‘special’ histological type, the disease is heterogeneous, and so identifying patients with poor prognostic subtypes will likely provide benefit in delineating more personalized and aggressive treatment or monitoring for disease progression. A detailed assessment of the genomic landscape of a large cohort of ILCs with long-term follow-up and/or in the context of treatment resistance will no doubt be essential to moving forward with precision medicine for patients diagnosed with this tumour type.

## Note

This article is part of a series on *Lobular breast cancer*, edited by Ulrich Lehmann. Other articles in this series can be found at http://breast-cancer-research.com/series/LBC.

## Abbreviations

aCGH: Array-based comparative genomic hybridisation
ALH: Atypical lobular hyperplasia
CGH: Comparative genomic hybridisation
DCIS: Ductal carcinoma *in situ*
EMT: Epithelial to mesenchymal transition
ER: Oestrogen receptor
IC-NST: Invasive carcinoma no special type
IDC: Invasive ductal carcinoma
ILC: Invasive lobular carcinoma
IntClust: Integrative cluster
LCIS: Lobular carcinoma *in situ*
LN: Lobular neoplasia
PLC: Pleomorphic lobular carcinoma
PLCIS: Pleomorphic lobular carcinoma *in situ*
TCGA: The Cancer Genome Atlas
